# Manipulating atmospheric CO_2_ concentration induces shifts in wheat leaf and spike microbiomes and in *Fusarium* pathogen communities

**DOI:** 10.3389/fmicb.2023.1271219

**Published:** 2023-10-10

**Authors:** Matthew G. Bakker, Briana K. Whitaker, Susan P. McCormick, Elizabeth A. Ainsworth, Martha M. Vaughan

**Affiliations:** ^1^Department of Microbiology, University of Manitoba, Winnipeg, MB, Canada; ^2^Mycotoxin Prevention and Applied Microbiology Research Unit, National Center for Agricultural Utilization Research, Agricultural Research Service, United States Department of Agriculture, Peoria, IL, United States; ^3^Global Change and Photosynthesis Research Unit, Agricultural Research Service, United States Department of Agriculture, Urbana, IL, United States

**Keywords:** *Fusarium*, mycotoxin, wheat, microbiome, FACE, global change, Fusarium head blight, phyllosphere

## Abstract

Changing atmospheric composition represents a source of uncertainty in our assessment of future disease risks, particularly in the context of mycotoxin producing fungal pathogens which are predicted to be more problematic with climate change. To address this uncertainty, we profiled microbiomes associated with wheat plants grown under ambient vs. elevated atmospheric carbon dioxide concentration [CO_2_] in a field setting over 2 years. We also compared the dynamics of naturally infecting versus artificially introduced *Fusarium* spp. We found that the well-known temporal dynamics of plant-associated microbiomes were affected by [CO_2_]. The abundances of many amplicon sequence variants significantly differed in response to [CO_2_], often in an interactive manner with date of sample collection or with tissue type. In addition, we found evidence that two strains within *Fusarium* – an important group of mycotoxin producing fungal pathogens of plants – responded to changes in [CO_2_]. The two sequence variants mapped to different phylogenetic subgroups within the genus *Fusarium*, and had differential [CO_2_] responses. This work informs our understanding of how plant-associated microbiomes and pathogens may respond to changing atmospheric compositions.

## 1. Introduction

We are currently experiencing a period of rapid global change, which is complicating efforts to predict and prepare for future agricultural challenges. In particular, the increasing concentration of atmospheric carbon dioxide (hereafter, [CO_2_]) from anthropogenic emissions is having complex effects on agricultural ecosystems. Progressively stronger impacts of increasing [CO_2_] on Earth’s energy budget and climate system are well known, but [CO_2_] also interacts contemporaneously with plant physiology in ways that may have implications for plant growth rate, water balance, disease dynamics, and other determinants of productivity (Long et al., 2006). The microbiome revolution has highlighted the myriad ways in which plant-associated microbes influence plant growth (Hawkes et al., 2021), but insufficient effort has been given to understanding how global change phenomena impact microbiomes of agricultural crops (Hacquard et al., 2022). Further study is needed to clarify how changing [CO_2_] affects multipartite plant-pathogen-microbiome interactions.

Plant-associated microbiomes are dynamic communities that change and develop over time. Therefore, changing environmental conditions (e.g., [CO_2_]) could influence microbial physiology and reproduction, which could subsequently impact microbial fitness or species interactions. For example, previous work showed that a dominant bacterial endophyte of soybean, *Methylobacterium*, was less abundant under elevated relative to ambient [CO_2_] conditions (Christian et al., 2021). One of the key microbiome functions of interest that we would like to understand, and ultimately to direct, is the ability to constrain the success of plant pathogens within the community (McLaren and Callahan, 2020). Thus, studies of pathogens as members of plant-associated microbiomes, under conditions that reflect key global change phenomena, are needed.

In practice, most experiments that manipulate [CO_2_] are not able to separate direct CO_2_ effects on microbes from effects that may be indirectly driven by the responses of plants to [CO_2_] (Whitaker and Bakker, 2019; Jin et al., 2020). However, it is likely that effects of [CO_2_] will be more pronounced for plants, which use CO_2_ as a substrate for photosynthesis, than for most microbes. Thus, additional clarity is needed regarding the extent to which plant responses to elevated [CO_2_] may interact with their associated microbiomes or with individual antagonistic symbionts (i.e., pathogens) of particular importance. For example, by alleviating limitations on the availability of carbon for fixation, elevated [CO_2_] permits plants to adjust stomatal conductance, increase water use efficiency, and increase biomass accumulation (Ainsworth and Rogers, 2007; Leakey et al., 2009). At the same time, longer durations of stomatal closure could reduce rates of infection by certain microbes, as stomates are important sites of ingress by pathogens and endophytes (Huang et al., 2018). Alternately, improved access to carbon may alter the chemical composition of plant tissue, potentially rendering it less nutritious or lowering the concentration of defensive compounds (Cuperlovic-Culf et al., 2019; Hay et al., 2020). Rates of plant development and timing of senescence may also be impacted (Gray and Brady, 2016), which may move plant-microbe interactions into different portions of the growing season, or affect the likelihood of confluence between exposure to inoculum and the presence of weather events that impact microbial establishment. Furthermore, greater availability of CO_2_ can impact carbon inputs to roots and soil (as in Lipson et al., 2005), which can lead to greater microbial biomass in soil (Liu et al., 2017), enhanced mycorrhizal symbiosis (Compant et al., 2010) and higher rates of respiration in soils under elevated [CO_2_] (Pendall et al., 2001). Previous research has demonstrated that elevated [CO_2_] can reduce the benefits provided by some fungal endophytes of grasses (Chen et al., 2017).

Wheat (*Triticum aestivum*) is a globally important cereal that responds to changes in [CO_2_]. Wheat grown at 550 ppm [CO_2_] produced 10.4% higher grain yield but with 7.4% lower grain protein, compared to growth at 380 ppm [CO_2_] (Högy et al., 2009). Concerningly, [CO_2_]-responsiveness of grain characteristics like protein content appears to be related to strength of resistance against the disease Fusarium head blight (FHB); i.e., cultivars of wheat that are more resistant to FHB also showed larger reductions in grain protein at elevated [CO_2_] (Hay et al., 2022). A persistent and damaging disease of small grain cereal crops, FHB is caused by several species within the genus *Fusarium*, and in North America primarily by *Fusarium graminearum* (McMullen et al., 2012). While there is yield loss associated with FHB, the accumulation of toxic fungal metabolites (e.g., deoxynivalenol [DON]) in the grain can be even more damaging (Bakker et al., 2018). Manipulative experiments can help determine the risk that this and other crop diseases will pose under a changing climate system. However, it is evident that disease management in the future will depend on how selected crop varieties, evolved pathogen populations, and the broader microbiome respond to changing environmental conditions (Váry et al., 2015).

In the present research, we performed two related experiments over two consecutive years to assess the impacts of elevated [CO_2_] on native microbial communities and Fusarium head blight disease risk in wheat. Wheat plants were grown in a Free Air Concentration Enrichment (FACE) system located in central Illinois (Aspray et al., 2023). The first experiment had the overall aim to test the impact of [CO_2_] on the wheat microbiome. We predicted that (1) elevated [CO_2_] would measurably impact the structure of plant-associated microbiomes, including via interactive effects with plant tissue type and collection date, which are already known to impact microbiome structure, and that (2) relative abundances of individual microbial taxa within the community would display significant changes in response to [CO_2_], collection date, and plant tissue type. (3) We also predicted that *Fusarium* spp. naturally found on the plants would have greater relative abundance on wheat heads than on leaves, would increase in relative abundance over time as disease progressed, and would respond to elevated [CO_2_]. In a second experiment, our aim was to assess Fusarium head blight disease development under ambient relative to elevated [CO_2_], in field conditions, using intentional inoculation with *F. graminearum*. In this experiment, we tested an additional hypothesis that (4) disease would vary between [CO_2_] treatments, based on previous reports that FHB symptoms can be more severe at elevated [CO_2_] under controlled conditions (Váry et al., 2015; Cuperlovic-Culf et al., 2019).

## 2. Materials and methods

### 2.1. Experimental field site

Research was conducted at the Soybean Free Air Concentration Enrichment (SoyFACE) facility (Aspray et al., 2023), where wheat was grown at ambient (~400 ppm) and elevated [CO_2_] (~600 ppm) for two growing seasons. Hard red spring wheat cultivar Glenn (moderately resistant to FHB; Mergoum et al., 2006) was hand planted in 2.7 m × 1.5 m plots (8 rows, 15 cm row spacing, 250 plants m^−2^) within the larger ambient and elevated [CO_2_] plots (each ~280 m^2^). The surrounding field and most of the area within the SoyFACE experimental plots were planted with soybean (*Glycine max*). All plots were rain watered and were not fertilized prior to planting. The SoyFACE experimental farm is operated as a maize-soybean rotation, where maize receives ~200 kg N ha^−1^ and the soybean does not receive N fertilizer (Aspray et al., 2023). Air temperature, relative humidity, and rainfall throughout the growing season were monitored onsite and at the Water and Atmospheric Resources Monitoring (WARM) station in Champaign^1^ and the Surface Radiation (SURFRAD) station (Aspray et al., 2023). Maximum and minimum temperatures during the June and July growing season were similar between years, while accumulated precipitation differed substantially (2017 = 110 mm, 2018 = 356 mm; Prism Climate Group at Oregon State University, 2023). In 2017, wheat was planted on June 6th, with *n* = 3 randomized blocks (one ambient and one elevated [CO_2_] plot per block). In 2018, wheat was planted on May 23rd, with *n* = 4 randomized blocks.

We performed two experiments within the SoyFACE site: (1) A profiling of the microbiome and of naturally present *Fusarium* spp. associated with wheat plants, and (2) An inoculation experiment, where *F. graminearum* strain Gz3639 was intentionally inoculated onto select wheat plants and disease progression was monitored.

### 2.2. Experiment 1: microbiome profiling and naturally present *Fusarium* spp.

#### 2.2.1. Plant sample collection and processing

In 2017, we harvested plant tissues for microbiome profiling beginning 1 week after anthesis and continuing at weekly intervals (24 July, 31 July, and 8 August). In 2018, sampling was expanded to a fourth timepoint, beginning at anthesis and extending for 3 weeks (10 July, 17 July, 24 July, and 31 July). On each sampling date, four plants were harvested per plot, by cutting the stem just above the soil surface. Plants were bagged individually and transported on dry ice to the laboratory, where they were stored at −20°C until further processing.

Tissue samples were placed into 50 mL aluminum grinding canisters, lyophilized for 2 days and homogenized to powder using metal ball bearings (0.95 cm diameter, 5 per tube) in a Geno/Grinder tissue homogenizer (SPEX SamplePrep) at 1650 rpm for 7 min. For samples from 2017, the spike and the flag leaf from each plant were separately processed. Thus, there were 144 tissue samples from 2017 (3 blocks × 2 [CO_2_] treatments × 3 collection dates × 2 tissue types × 4 individual plants). For samples from 2018, we adjusted the tissue processing method to ensure that a standard quantity of tissue was available in every case; spikes and flag leaves were bulked across plants within a plot, homogenized, and subsampled in duplicate. Thus, there were 128 tissue samples from 2018 (4 blocks × 2 [CO_2_] treatments × 4 collection dates × 2 tissue types × 2 subsamples).

DNA extractions were performed on 20 mg of pulverized tissue, except for the 2017 flag leaf samples which were processed in their entirety, due to low biomass (minimum 16.7 mg). Extracts of DNA were diluted to 5 ng μL^−1^. Generation of amplicons for sequencing was accomplished using a two-stage PCR. For bacteria in 2017, we targeted the v5-v6 region of the 16S ribosomal RNA (rRNA) gene, using primers 779F (Chelius and Triplett, 2001) and the reverse complement of 1114F (Lundberg et al., 2012). In 2018, we targeted the v4 region using primers 515F and 806R (Caporaso et al., 2011), in order to reduce the abundance of chimeric sequences seen in 2017 (12.2% of reads). For fungi in 2017 we targeted the first internal transcribed spacer (ITS1) using primers ITS1f and ITS2 (Smith and Peay, 2014); while in 2018 we targeted the second internal transcribed spacer (ITS2) using primers ITS3_KYO2 and ITS4_KYO3 (Toju et al., 2012) to improve differentiation among *Fusarium* spp. (Bakker, 2018). Negative controls were included in the amplicon sequencing libraries, by performing blank DNA extractions and performing PCR with no template DNA. Mock communities of known composition and structure were used as reference samples. The bacterial mock community was catalog item MSA-1003 from the American Type Culture Collection, while the fungal mock community was from Bakker (2018).

Each PCR mixture consisted of 0.5 U Phusion High-Fidelity DNA Polymerase and associated Phusion Green HF buffer (ThermoFisher), dNTPs at 200 μM final concentration, upstream and downstream primers each at 0.5 μM final concentration, 2.5 μL of template DNA, and nuclease-free water to 25 μL per reaction. Thermal cycling consisted of: 98°C for 30 s; 25 cycles of: 98°C for 10 s, 55°C for 30 s, 72°C for 15 s; and a final extension step at 72°C for 5 min. Amplicons were purified using AMPure XP beads (Beckman Coulter). Sample-specific barcodes were added to the amplicons via a second PCR step, using the Nextera XT Index Kit (Illumina) according to the manufacturer’s protocol except that we used Phusion High-Fidelity DNA Polymerase. Thermal cycling was as for amplicon generation, except that only 8 cycles were performed. Indexed amplicons were bead cleaned as described previously, and DNA concentration was determined via the Quant-iT dsDNA Assay Kit (Invitrogen), using a qPCR instrument (BioRad CFX96) to measure fluorescence. Indexed amplicons were pooled in equimolar ratios.

After pooling, libraries were analyzed on a TapeStation instrument (Agilent) for assessment of amplicon size distribution and concentration. Libraries were then size-selected via gel recovery (Lonza FlashGel) to remove probable primer dimers. In total, three libraries were sequenced using a MiSeq instrument (Illumina): library 1 consisted of bacterial plus fungal amplicons from 2017 samples (v2 500 cycle sequencing kit), library 2 consisted of bacterial amplicons from 2018 samples (v2 500 cycle), and library 3 consisted of fungal amplicons from 2018 samples (v3 600 cycle). Raw sequence data, as output by the MiSeq software, are available in the NCBI Sequence Read Archive (BioProject PRJNA544326).

#### 2.2.2. Bioinformatics of microbiome sequence data

Amplicon sequences were processed in R v.4.0.2 (R Core Team, 2022) using DADA2 (Callahan et al., 2016). Briefly, primer sequences were removed using Cutadapt (Martin, 2011). Reads were filtered to permit a maximum of 2 expected errors (Edgar and Flyvbjerg, 2015), trimmed at the 3′ ends to remove low quality bases, error-corrected, and denoised. Then, forward and reverse reads were merged, permitting up to one mismatch in the overlapping region, and chimeras removed. The DADA2 output yields Amplicon Sequence Variants (ASVs), or clusters of sequencing reads that differ by as little as 1–2 single nucleotide polymorphisms (Callahan et al., 2017). Lastly, the ITS datasets were additionally processed through ITSx v.1.1.2 (Bengtsson-Palme et al., 2013) to trim off conserved ribosomal RNA gene regions flanking the ITS, as well as to flag ASVs of likely non-fungal origin for removal.

Putative taxonomic assignments for ASVs were made using a naïve Bayesian classifier (Wang et al., 2007), with the Silva reference alignment v.138 (Quast et al., 2013) for the bacterial dataset and the UNITE database v.8.2 (Kõljalg et al., 2013) for the fungal dataset. Within the bacterial datasets, ASVs assigned to chloroplast or mitochondria were culled. Negative control samples were used to identify putative contaminants (Davis et al., 2018); 24 ASVs were removed from the biological samples as probable contaminants. Mock community controls were processed along with the biological samples. The resulting sample-by-ASV abundance tables are provided in the Zenodo digital repository (Whitaker and Bakker, 2023).

#### 2.2.3. Statistical analyses of experiment 1

Manipulation and analyses of the processed microbiome data primarily occurred using the packages phyloseq v.1.40.0 (McMurdie and Holmes, 2013) and DESeq2 v.1.36.0 (Love et al., 2014). Analyses were performed separately by kingdom (bacteria = ‘B’, fungi = ‘F’) and all contrasts were made within years (2017 = ‘17’, 2018 = ‘18’) and not between years, because aspects of sample processing were confounded by year.

To address our first hypothesis about the response of the wheat microbiome to [CO_2_], collection date, and plant tissue type, we assessed microbiome community structure and observed richness using a linear model framework. Both microbiome structure and taxon richness were modeled using a series of linear models via residual randomization in a permutation procedure (package RRPP v.1.3.0; 1,000 permutations; Collyer and Adams, 2018). The RRPP package allows for the analysis of complex mixed model designs via the explicit selection of denominators for F-ratio calculations, as well as the ability to use Type III sum of squares in pseudo-F statistic calculations. We tested [CO_2_], collection date, and plant tissue type as fixed effects, and the nested factor of block into [CO_2_] as a random effect, along with all statistical interaction terms. The denominators for F-ratio calculations were chosen following rules specified by Underwood (1996) and are indicated in the supplemental tables of raw model results (Supplementary Tables S1, S2). For the microbiome structure analyses, the response variable was a Euclidean distance matrix calculated from the variance-stabilized ASV abundance table (DESeq2), after the removal of infrequent and low abundance taxa (i.e., ASVs found fewer than 5 times in less than 10 samples were removed prior to analysis). This distance matrix was used to visualize patterns in community structure, via principal coordinate ordinations, which were split by year, microbial kingdom, and experimental treatments as necessary to explore significant treatment interactions. For the 2018 datasets, we also constructed trajectory plots using the mean principal coordinates of community distances (De Cáceres et al., 2019), to better display changes in community structure between [CO_2_] treatments across collection dates.

To address our second hypothesis and identify specific microbial taxa that responded to [CO_2_], collection date, plant tissue type, and the interactions among these factors, we performed a differential abundance analysis using DESeq2 (Love et al., 2014). Briefly, abundances (i.e., raw counts of sequences observed per ASV) were modeled as the response, using negative binomial generalized linear models. The base model included the main fixed effects of [CO_2_], collection date, and tissue type, as well as the nested random effect of the Block × CO_2_ interaction term. To assess the significance of differential abundances across main effects, the deviance of the full base model was compared to a reduced model lacking the effect being tested (as in Wagner et al., 2016). For example, to test for response of ASV abundance to [CO_2_], we compared the base model described above to a reduced model containing only Collection + Tissue + Block × CO_2_. To assess whether two-way interactions among experimental factors (i.e., CO_2_ × Collection, CO_2_ × Tissue, or Collection × Tissue) were significant in predicting microbial ASV abundances, we compared deviance of the base model to the base model plus the interaction term of interest. Using Wald Tests, we estimated the log_2_-fold change in abundance for each ASV across all pairwise contrasts within the treatment variables (for example, between any given two collection points or between tissue types within a [CO_2_] level). Significance was determined at *p* < 0.05 after adjustment for multiple comparisons using the Benjamini-Hochberg false discovery rate (BH-FDR). We performed a second *p*-value adjustment using the BH-FDR for each term in the model which required greater than two pairwise comparisons (i.e., collection date and all two-way interactions), because in these cases multiple Wald Tests were conducted on each ASV.

To address our third hypothesis about the responsiveness of naturally-occurring *Fusarium* populations to [CO_2_], we examined the [CO_2_] responsiveness of all ASVs from the DESeq2 results that were assigned to the genus *Fusarium*. In 2017, no ASVs assigned to *Fusarium* were responsive to [CO_2_] treatments. In 2018, 10 ASVs that were assigned to the genus *Fusarium* were significantly responsive to the treatment factors (DESeq2). To assign *Fusarium* ASVs to species complexes within the genus (O'Donnell et al., 2015), we aligned the ASV sequences together with sequences of reference strains *F. graminearum* PH1 (NCBI accession NC026477) and *F. incarnatum* NRRL 13379 (NCBI accession GQ505680). A maximum likelihood phylogeny was constructed using default parameters of CLC Genomics Workbench software v. 23.0.1 (Qiagen), with the Jukes Cantor model of nucleotide substitution.

### 2.3. Experiment 2: deliberate inoculation with a pathogen

#### 2.3.1. Inoculation with *Fusarium graminearum*

Inoculum of *F. graminearum* strain Gz3639 was prepared by transferring two mycelia plugs of actively growing culture on V8 media plates into 20 mL of mung bean broth. The culture was grown in the dark at 28°C and 200 rpm for 3 days. The macroconidia were then pelleted twice, rinsed, and resuspended in sterile 0.04% Tween 20. The spores were counted using a hemocytometer and adjusted to a concentration of 1 × 10^5^ macroconidia mL^−1^ in 0.04% Tween 20. In 2017, 30 flowering wheat spikes (Feekes 10.5.2; 17 July) per plot were inoculated by pipetting 10 μL of the spore suspension between the palea and lemma of a central floret on each spike. The inoculated spikes were on completely different plants than those that were sampled for microbiome profiling. In 2017, the inoculated/diseased spikes experienced bird and field mice herbivory at greater rates than surrounding uninoculated plants. Thus, all the remaining inoculated spikes (7 spikes from ambient [CO_2_] plots and 5 spikes from elevated [CO_2_] plots) were collected at 14 days post inoculation (31 July). In 2018, 50 flowering wheat spikes (Feekes 10.5.2; 10 July) were selected per plot and inoculated with *F. graminearum*. Fourteen inoculated spikes were collected per plot on each of 7, 14, and 21 days after *F. graminearum* inoculation (17 July, 24 July, and 31 July, respectively).

#### 2.3.2. Quantification of disease progression

Harvested wheat spikes were transported to the laboratory on dry ice where they were lyophilized and ground, as in Experiment 1. Subsamples of pulverized tissue (20 mg) were extracted for DNA using the PureLink Plant Total DNA Purification Kit (Invitrogen). DNA extracts were diluted to 5 ng μL^−1^ (Nanodrop). The density of *F. graminearum* within each wheat spike was measured using quantitative polymerase chain reaction (qPCR). To minimize error associated with variation in DNA extraction efficiencies, *F. graminearum* DNA abundance was expressed relative to wheat DNA abundance (hereafter ‘*Fusarium* load’), as has been done in other plant-fungal interaction studies (e.g., Bodenhausen et al., 2021). In 2017, we assessed the *F. graminearum* Tri6 and wheat PR1 genes using the primers and methods reported in Taylor et al. (2022). Specifically, *Fusarium* load was calculated using the 2^ΔCq^ method, where ΔCq is the difference between the arithmetic mean of three technical replicate Cq values from the wheat vs. the *F. graminearum* assay.

For samples from 2018, we increased the number of qPCR assays to improve the reliability of our measurements, targeting three different *F. graminearum* genes and three different wheat genes. Assays for *F. graminearum* genes RED, TEF, and Tri101 and for wheat genes Actin and PAL were reported in Taylor et al. (2022). We added an assay, designed in this work, for the wheat translation elongation factor 1-α gene, consisting of: upstream primer (GAT TGA CAG GCG ATC TGG TAA G), probe (TCC TCA AGA ATG GTG ATG CTG GCA; 5′ 6-FAM/ZEN/3′ IBFQ), and downstream primer (GGC TTG GTG GGA ATC ATC TT). The qPCR was run using the Juno and Biomark HD systems (Fluidigm) with the 192.24 Dynamic Array integrated fluidic circuit and following the manufacturer’s protocol (Fluidigm PN 100–6,174), with TaqMan Fast Advanced Master Mix (Life Technologies). Four technical replicates were run per each sample-assay combination. Raw fluorescence values at each PCR cycle were processed through the LinRegPCR data analysis program (Ramakers et al., 2003), which assesses the amplification efficiency for each reaction and calculates a starting quantity, N_0_. To summarize these data, we defined:


$$\mathrm{Fusarium}\;\mathrm{load}=\frac{\mathrm{geometric}\;\mathrm{mean}\left(\begin{array}{l} N_{0\_Fg\_\mathrm{RED}},\;N_{0\_Fg\_TEF},\;\\ \;N_{0\_Fg\_Tri101} \end{array}\right)}{\mathrm{geometric}\;\mathrm{mean}\left(\begin{array}{l} N_{0\_Ta\_\mathrm{Actin}},\;N_{0\_Ta\_Ef1},\;\\ \;N_{0\_Ta\_\mathrm{PAL}} \end{array}\right)}$$

In both 2017 and 2018, pulverized spike tissue was also subsampled for determination of DON concentration (hereafter, [DON]). Ground tissue (targeting 0.5 g) was extracted with 10 mL acetonitrile and water (86:14). 5 mL of each extract was purified with a MycoSep 225 Trich cartridge (Romer Labs); 2 mL of the purified extract was dried under a stream of nitrogen. Trimethylsilyl (TMS) derivatives were prepared by adding 100 μL of a 100:1 freshly prepared mixture of 1-(trimethylsilyl) imidazole/trimethylchlorosilane to the dried extracts. 900 μL isooctane and 1 mL water were then added to each sample and the mixtures were gently vortexed until clear. The top isooctane layer was transferred to a GC vial and analyzed by GCMS. TMS derivatives of purified DON were prepared in the same way and used to construct a standard curve (0.3125 μg to 80 μg). DON concentrations were determined with GC–MS with a splitless inlet and selective ion monitoring for the triTMS-DON. The GC oven was at 150°C at injection and held at 150°C for 1 min before heating to 280°C at 30°C min^−1^ and then held at 280°C for 3.5 min. TriTMS-DON was detected at 6.2 min.

#### 2.3.3. Statistical analyses of experiment 2

To address the hypothesis regarding the impact of elevated [CO_2_] on FHB development following intentional inoculation with *F. graminearum*, we analyzed the data for each year separately. For the 2017 data, we constructed linear models with [CO_2_] as a fixed effect. For the more robust 2018 dataset, we constructed mixed effects models using lme4::lmer v.1.1.30 (Bates et al., 2015) with Block × CO_2_ as the random effect, and fixed effects of [CO_2_], days post infection, and their interaction. The response variables tested in both years were *Fusarium* load and [DON], which were transformed as necessary to meet assumptions of normality and homoscedasticity.

## 3. Results

### 3.1. Overview of microbiome diversity

Microbiome profiling identified a diverse set of microbial taxa across years and kingdoms. After removal of chimeras, sequences of non-microbial origin, and contaminants, there were 552 bacterial and 165 fungal ASVs in 2017, and 654 bacterial and 1,375 fungal ASVs in 2018. However, these wheat-associated microbiomes were dominated by a small number of ASVs. In 2017, the top 10 most abundant ASVs accounted for 75.0% of the total observed reads in the bacterial dataset and 96.3% of the fungal dataset. In 2018, the top 10 most abundant ASVs accounted for 63.9% of the bacterial and 79.7% of the fungal dataset. The majority of bacterial ASVs belonged to the phyla Proteobacteria, Actinobacteria, Firmicutes, and Bacteroidota, while the majority of fungal ASVs belonged to the phyla Ascomycota and Basidiomycota. Mock community reference samples demonstrated that our approach provided a reasonable approximation of true microbiome composition and structure; Bray–Curtis similarity values for the contrast of observed vs. expected mock community profiles were 0.653 ± 0.00068 (2017 bacteria), 0.792 ± 0.0028 (2017 fungi), 0.646 ± 0.017 (2018 bacteria), and 0.817 ± 0.0055 (2018 fungi).

### 3.2. Responsiveness of microbiome structure and richness to [CO_2_], collection date and tissue type

Tissue type (flag leaf vs. spike) was a key driver of differences in microbiome community structure across years and microbial kingdoms (Supplementary Figure S1). However, despite the clear visual separation in community structure between the two tissue types in both years, many of the most abundant ASVs were present in both tissue types. Specifically, 87.7% (2017 bacteria), 87.5% (2017 fungi), 80.7% (2018 bacteria), and 95.0% (2018 fungi) of the most abundant ASVs were shared between flag leaf and spike habitats. Thus, differences in microbial community structure between the two wheat tissues primarily reflected altered microbial abundance, rather than indicating differential microbial presence and absence.

**Figure 1:**
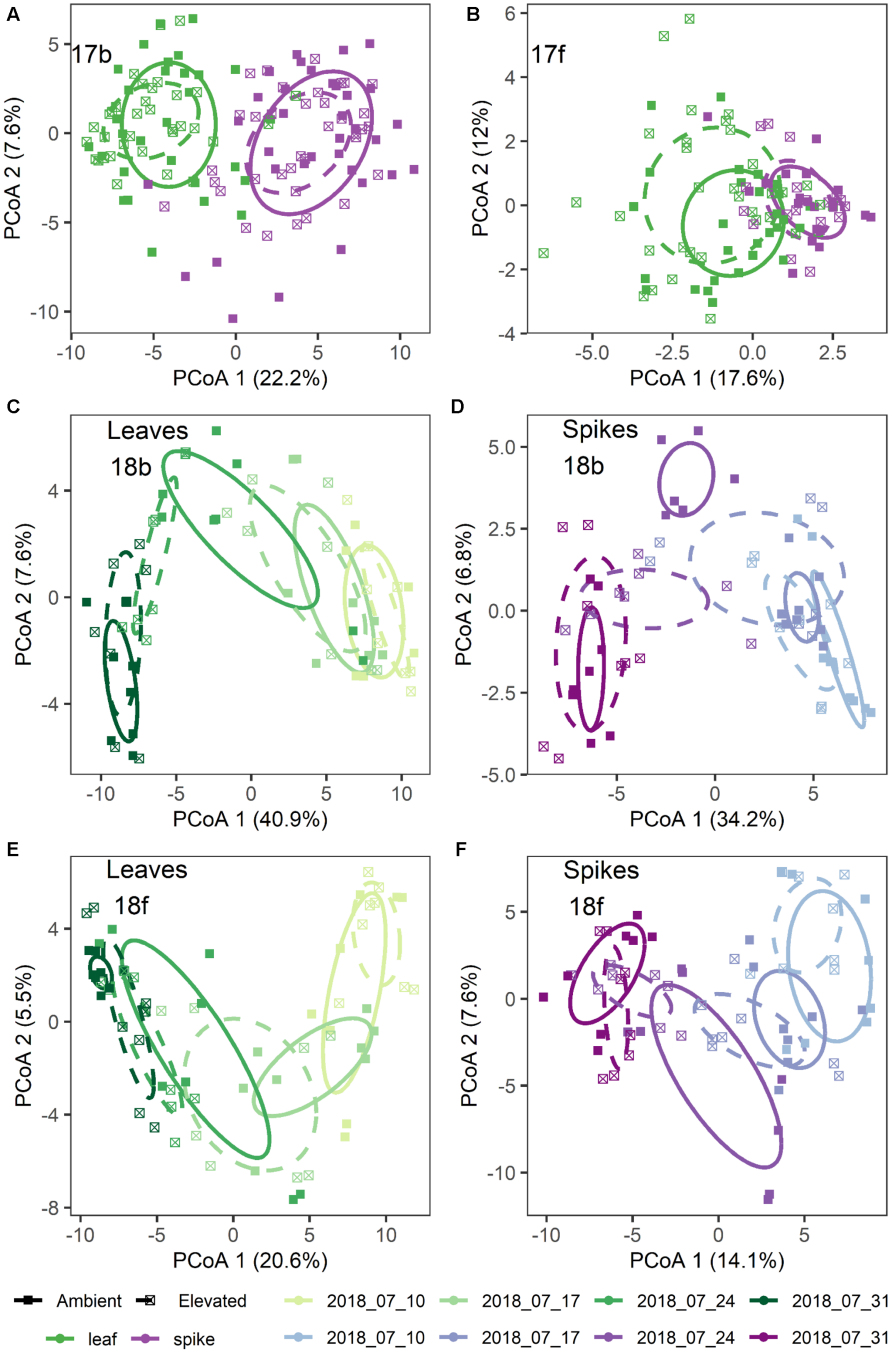
Microbiome structural differences by treatment for: **(A)** 2017 bacteria; **(B)** 2017 fungi; **(C,D)** 2018 bacteria; and **(E,F)** 2018 fungi. Shown are principal coordinates ordinations based on pairwise Euclidean distances calculated from a table of variance stabilized abundances of amplicon sequence variants. Each point represents a single sample. Ellipses represent one standard error around the centroid for each treatment group. Colors denote tissue type **(A,B)** and color shades denote collection date **(C–F)**. Leaf tissues are shown in green shades **(C,E)** and spike tissues are shown in purple shades **(D,F)**. In all panels, point shapes and line types denote [CO_2_] treatment.

Additionally, [CO_2_] and date of sample collection modified the effect of tissue type on the microbiome, but these effects were highly dependent on sampling year and to a lesser extent microbial kingdom (Supplementary Table S1). For example, in 2017 bacterial communities were structured by a significant three-way interaction between [CO_2_], tissue type, and the nested random effect of Block × [CO_2_] (*p* = 0.045; Figure 1A), while fungal communities were structured by a significant four-way interaction between [CO_2_], collection date, tissue type, and the nested random effect of Block × [CO_2_] (*p* = 0.024; Figure 1B; Supplementary Table S1). Visual inspection of community structure changes revealed that the flag leaf communities (and particularly fungal communities on flag leaves) were more strongly affected by [CO_2_] treatment than were the spike communities (Figures 1A,B). In addition, there was also a significant main effect of [CO_2_] treatment on the bacterial communities (*p* = 0.010; Figure 1A), but not on the fungal communities (*p* = 0.78).

**Figure 2:**
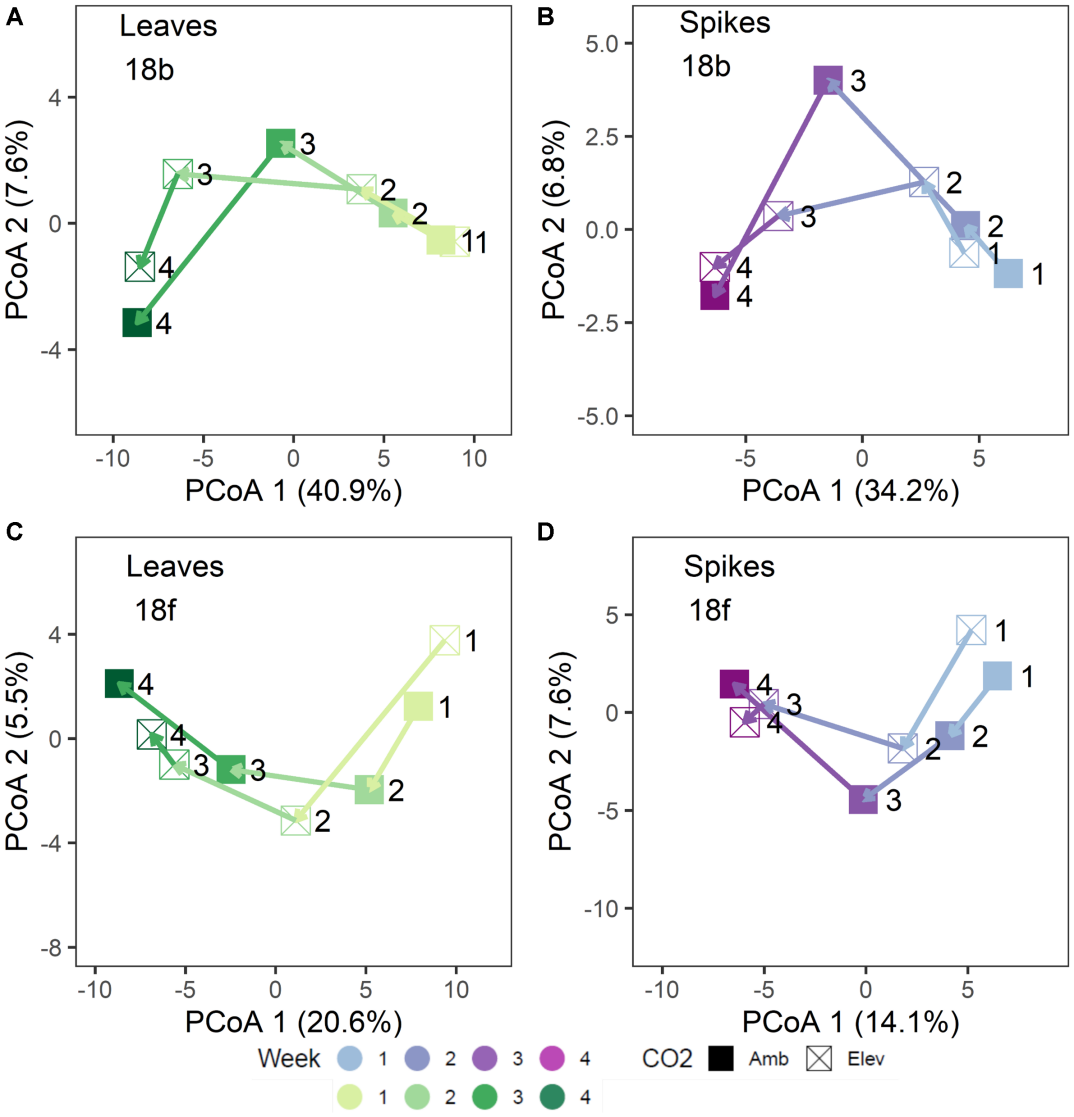
Trajectory plots of 2018 microbiome community distances: **(A)** bacterial leaf, **(B)** bacterial spike, **(C)** fungal leaf, and **(D)** fungal spike microbiomes. Each point represents the mean principal coordinates of community distance for each [CO_2_] x Collection Week treatment group. Point shapes denote [CO_2_] treatment. Arrows denote mean treatment trajectory. Colors shades denote collection date. Leaf tissues are shown in green shades **(A,C)** and spike tissues are shown in purple shades **(B,D)**. For clarity, each point is labeled with the collection week number (week 1 – week 4).

In 2018, separation of the community structure data by tissue type revealed a clear pattern of microbiome change over collection date (Figures 1C–F). Specifically, bacterial community structure changed over the course of wheat flowering and seed set (i.e., by collection date; *p* = 0.001), contingent on the interaction with tissue type (*p* = 0.035; Figures 1C,D). The impact of bacterial community change over time was also modified in part by a significant interaction with [CO_2_] (*p* = 0.006; Figures 1C,D). For fungal communities, the impact of tissue type on community structure was moderated by a significant three-way interaction of tissue type × [CO_2_] × collection date (*p* = 0.027; Figures 1E,F), as well as by the interaction of collection date × tissue type (*p* = 0.001), and by a main effect of collection date (*p* = 0.004). Trajectory plots constructed using the mean principal coordinates of community distances, showed that [CO_2_] treatment had particularly pronounced effects on bacterial spike, fungal spike, and bacterial leaf communities 3–4 weeks after anthesis (Figures 2A–D). Specifically, the elevated [CO_2_] communities 3 weeks after anthesis were more similar to the ambient [CO_2_] communities 4 weeks after anthesis, as can be seen by evaluating the leftward shift in the average microbiome profile along the principal coordinates axis 1 (Figures 2A–D).

Elevated [CO_2_] conditions often decreased the observed microbial richness, but the magnitude of the reduction varied by year and microbial kingdom, as well as interacting significantly with other experimental treatments (see Supplementary Table S2 for full ANOVA results). In 2017, bacterial richness was moderately affected by the nested random effect Block × [CO_2_] (*p* = 0.027; Supplementary Figure S2A), while fungal richness varied by a significant three-way interaction of [CO_2_] × tissue type × block (*p* = 0.031; Supplementary Table S2; Supplementary Figure S2B). Specifically, fungal richness in 2017 decreased under elevated [CO_2_] in spike tissues but not in flag leaves. In 2018, bacterial richness varied by the interaction of [CO_2_] × collection date (*p* = 0.025), as well as by a main effect of collection date (*p* = 0.007; Supplementary Table S2; Supplementary Figure S2C). As in 2017, fungal richness in 2018 was more dependent on tissue type. Specifically, fungal richness was influenced by a significant three-way interaction between [CO_2_] × tissue type × block (*p* = 0.001; Supplementary Table S2; Supplementary Figures S2D,E). In other words, fungal richness in 2018 was reduced under elevated [CO_2_] in both leaf and spike tissues, but the magnitude of the effect varied by block (Supplementary Figures S2D,E).

### 3.3. Differential abundance of individual microbial taxa across treatments

Differential abundance analysis clarified the responsiveness of individual ASVs to experimental factors and provided a more detailed understanding of the observed shifts in overall community structure (Supplementary Tables S3, S4). Most of the statistically significant enrichments or depletions in the abundance of individual ASVs occurred across collection dates, either as a main effect (630 significantly responsive ASVs) or in interaction with either differential response to tissue types (1092) or differential response to [CO_2_] treatment (381; Table 1). As main effects, tissue type (spike vs. flag leaf) induced significant changes in the abundances of 242 ASVs, while [CO_2_] treatment induced significant changes in the abundances of 61 ASVs (Table 1).

**Table 1:** The abundances of many amplicon sequence variants (ASVs) were significantly impacted by experimental factors.

	Number of ASVs tested	[CO_2_] (ambient vs. elevated)	Collection (3 dates in 2017, 4 dates in 2018)	Tissue (spike vs. flag leaf)	[CO_2_] × Collection	[CO_2_] × Tissue	Collection × Tissue
2017 bacteria	73	13	28	53	46	32	55
2017 fungi	16	0	3	5	5	2	2
2018 bacteria	135	12	299	82	176	80	546
2018 fungi	180	36	300	102	154	88	489
Sum	404	61	630	242	381	202	1092

Shown are counts of significant responses to each treatment factor (i.e., unique responses to all pairwise combinations within each treatment factor, including interactions). See methods for full statistical details. The total number of ASVs tested is included as a reference for the size of each microbiome dataset after filtering out infrequent or low abundance taxa.

**Figure 3:**
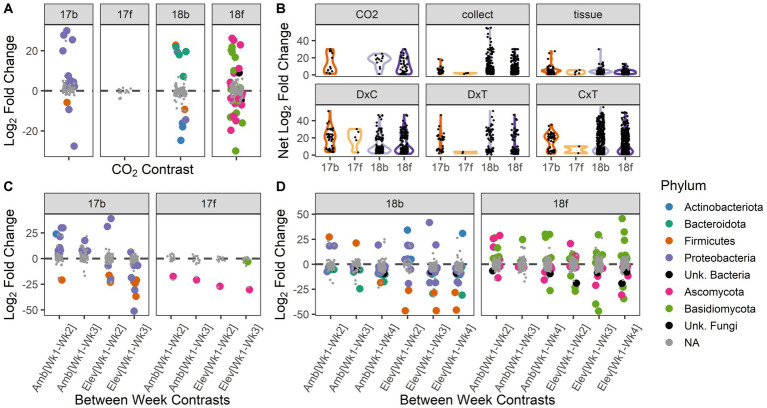
Abundances of individual microbial taxa respond to experimental treatments and interactions among treatments. Differential abundance results depicted according to year and microbial kingdom: bacteria and fungi in 2017 (17b, 17f) and 2018 (18b, 18f), respectively. **(A)** The abundances of individual microbial taxa are responsive to [CO_2_] treatment. Positive values indicate enriched abundance under ambient vs. elevated [CO_2_], and vice versa for negative values. **(B)** Change in abundance (net log2 fold change) for taxa that responded significantly to experimental factors. Violin plots show the range of the data and relative distribution. ‘CO_2_’ = ambient vs. elevated concentration of carbon dioxide; ‘collect’ = collection date (3 dates in 2017, 4 dates in 2018), ‘tissue’ = spike vs. flag leaf, ‘D × C’ = interactions between carbon dioxide treatment and collection date, ‘D × T’ = interactions between carbon dioxide treatment and tissue type, ‘C × T’ = interactions between collection date and tissue type. **(C)** Individual microbial taxa respond to the interaction of [CO_2_] and collection date in 2017. Positive values indicate a decrease in relative abundance over time within that [CO_2_] treatment, and vice versa for negative values. **(D)** Individual microbial taxa respond to the interaction of [CO_2_] and collection date in 2018. Positive values indicate a decrease in relative abundance over time within that [CO_2_] treatment, and vice versa for negative values. For panels **(A,C,D)**: statistically significant pairwise contrasts are color coded by microbial phylum; non-significant effects are shown in gray.

Despite the relatively few microbial ASVs that responded to [CO_2_] treatment as a main effect, those ASVs that were significantly responsive to [CO_2_] exhibited large shifts in abundance (overall median log_2_-fold change among [CO_2_] treatments = 11.1; for 2017 bacteria = 7.5; 2018 bacteria = 18.0; 2018 fungi = 9.5; Figure 3B). Similarly, those ASVs that were significantly impacted by the interaction of collection date × [CO_2_] exhibited large changes in abundance (overall median log_2_-fold change = 8.0; for 2017 bacteria = 20.1; 2017 fungi = 20.7; 2018 bacteria = 7.5; 2018 fungi = 7.8; Figure 3B). Individual microbial ASVs were next most responsive to the interaction of collection date × tissue type (overall median log_2_-fold change = 7.6; for 2017 bacteria = 20.1; 2017 fungi = 6.1; 2018 bacteria = 7.2; 2018 fungi = 7.9; Figure 3B). For taxonomic information (i.e., phylum) about the microbiota responding to each treatment see Supplementary Figure S3.

Microbial taxa that are responsive to [CO_2_] may have important implications for future management of microbiota in crops. Thus, we chose to assess the ASVs showing differential abundance across [CO_2_] treatments more closely. In 2017, 13 bacterial ASVs responded to [CO_2_], with the majority being enriched under ambient conditions, and many belonging to the phylum Proteobacteria (Figure 3A). Interestingly, many ASVs belonging to phylum Proteobacteria were depleted over time under ambient [CO_2_] conditions but enriched over time under elevated [CO_2_] conditions (Figure 3C). ASVs belonging to phylum Firmicutes were typically enriched over time but were more responsive to elevated than ambient [CO_2_] conditions (Figure 3C). No fungal ASVs were differentially enriched or depleted between ambient and elevated [CO_2_] conditions in 2017 (Figure 3A). However, two fungal ASVs were enriched over time conditional on [CO_2_] treatment, including a *Sporobolomyces* sp. (phylum Basidiomycota), which was only enriched over time under elevated [CO_2_] conditions, and a *Bipolaris* sp. (phylum Ascomycota; Figure 3C).

In 2018, the abundances of 12 bacterial ASVs spanning four phyla were significantly impacted by [CO_2_], with half enriched in each [CO_2_] treatment (Figure 3A). Thirty-five bacterial ASVs were differentially enriched or depleted across collection dates conditional on [CO_2_] treatment (Figure 3D). The majority of affected ASVs belonged to the phylum Proteobacteria. However, two ASVs belonging to phylum Firmicutes (family Paenibacilliaceae) showed the greatest median log_2_-fold change overall and showed a weaker enrichment over time in ambient relative to elevated [CO_2_] (Figure 3D).

In 2018, the abundances of 36 fungal ASVs were significantly impacted by [CO_2_] treatment (Figure 3A). The median log_2_-fold change of Basidiomycota ASVs under [CO_2_] conditions was 2.8× greater than for Ascomycota ASVs (Figure 3A). The magnitude of responsiveness to collection date (median log_2_-fold change) was 1.3× higher under elevated [CO_2_] than under ambient [CO_2_] conditions (Figure 3D). For both the bacterial and fungal kingdoms in 2018, the largest changes in microbial abundance under ambient [CO_2_] occurred during weeks 2–3, while under elevated [CO_2_] the largest changes in microbial abundance occurred during weeks 3–4 (Supplementary Tables S3, S4). In sum, the differential abundance analysis of individual ASVs in 2018 was reflective of the shift in microbiome trajectory noted in the overall community structure results (Figures 2A–D).

### 3.4. Responses of naturally present *Fusarium* populations to elevated [CO_2_]

**Figure 4:**
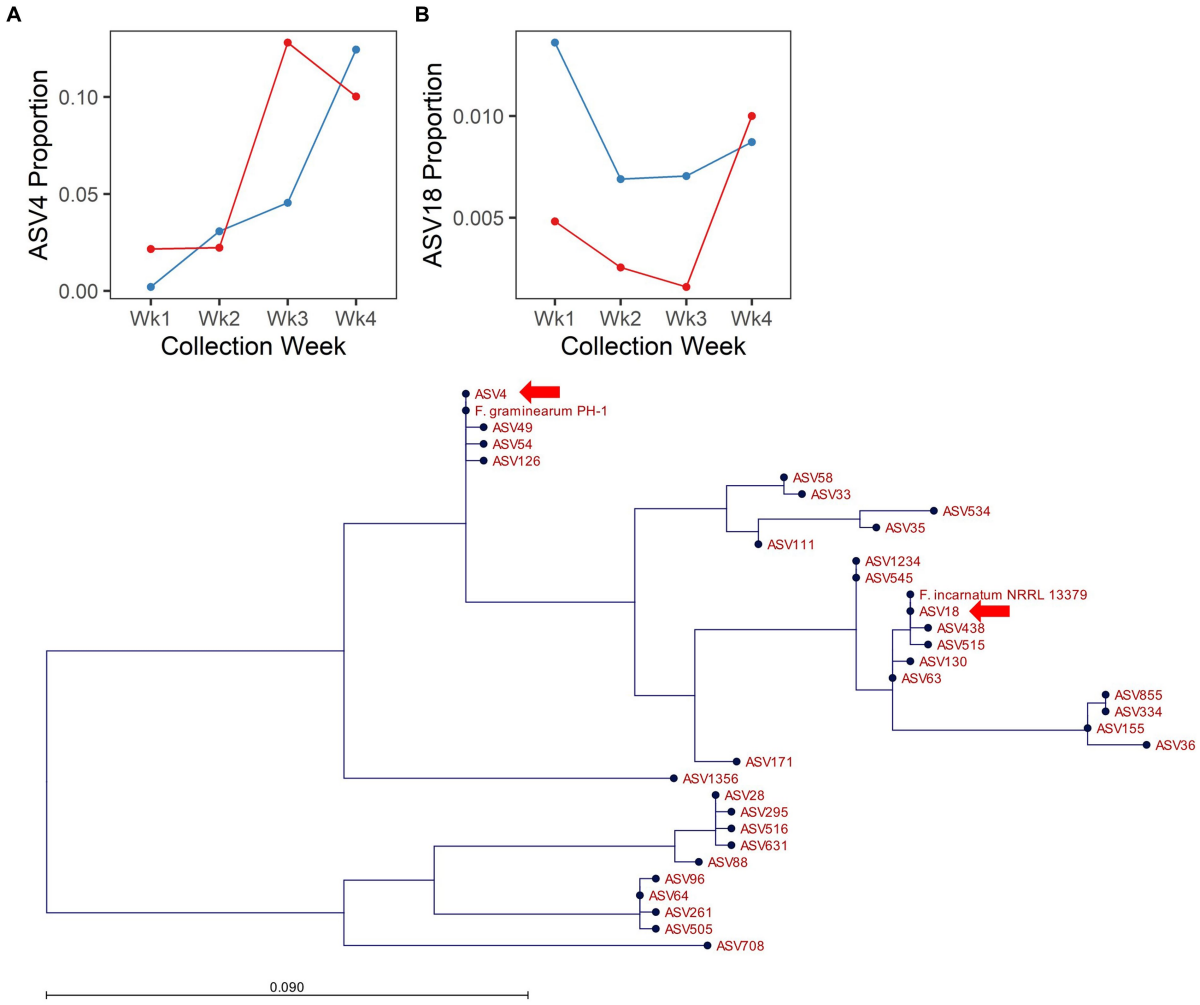
Endogenous *Fusarium* spp. respond differentially to [CO_2_] treatment in 2018. Line plots depict the proportion of cumulative sequencing read abundance attributed to specific amplicon sequence variants assigned to *Fusarium*: **(A)** ASV4 and **(B)** ASV18, according to [CO_2_] treatment (blue = ambient, red = elevated) and collection week. **(C)** Depicts phylogenetic placement of ASV4 and ASV18 (arrows), with respect to reference strains and other ASVs assigned to *Fusarium*.

*Fusarium* is an important global pathogen of wheat and was highlighted here as an indicator of disease risk (i.e., Fusarium head blight). In 2017, no ASVs assigned to the genus *Fusarium* were responsive to [CO_2_]. However, in 2018, several ASVs assigned to the genus *Fusarium* were responsive to the interaction of [CO_2_] × collection date (ASV4, ASV58, ASV63, ASV64) or to the main effect of [CO_2_] (ASV18, ASV33, ASV58, ASV88; Supplementary Table S4). Of these, ASV4 and ASV18 were the most abundant (ASV4 = 5.86% and ASV18 = 0.72% of filtered fungal sequences from 2018) and were also more abundant in spike relative to flag leaf tissue (10.5× and 2.8× more abundant in the spike vs. flag leaf for ASV4 and ASV18, respectively; Supplementary Figure S4), suggesting they may have a potential role in FHB. Inclusion of reference sequences in a maximum likelihood phylogeny of our observed *Fusarium* amplicon sequences highlighted that ASV4 was identical to the corresponding amplicon from *F. graminearum* PH-1, and ASV18 was identical to the corresponding amplicon from *F. incarnatum* NRRL 13379 (Figure 4C). Although the ITS region is not an ideal taxonomic marker for the genus *Fusarium*, it is considered sufficient for identification to the level of species complex (O'Donnell et al., 2022). Thus, ASV4 likely represents *F. graminearum* or a closely related species from the *Fusarium sambucinum* species complex, while ASV18 likely represents a species from the *Fusarium incarnatum-equiseti* species complex. As expected for a putative pathogen establishing on a host plant, ASV4 increased in abundance over time. However, enrichment of this ASV was more rapid under elevated relative to ambient [CO_2_] (Figure 4A). In contrast, across all collection dates, ASV18 was 18.7× more abundant under ambient relative to elevated [CO_2_] conditions (Figure 4B). Thus, the effects of elevated [CO_2_] were not consistent across members of the genus *Fusarium*.

### 3.5. Response of inoculated *Fusarium graminearum* to [CO_2_]

**Figure 5:**
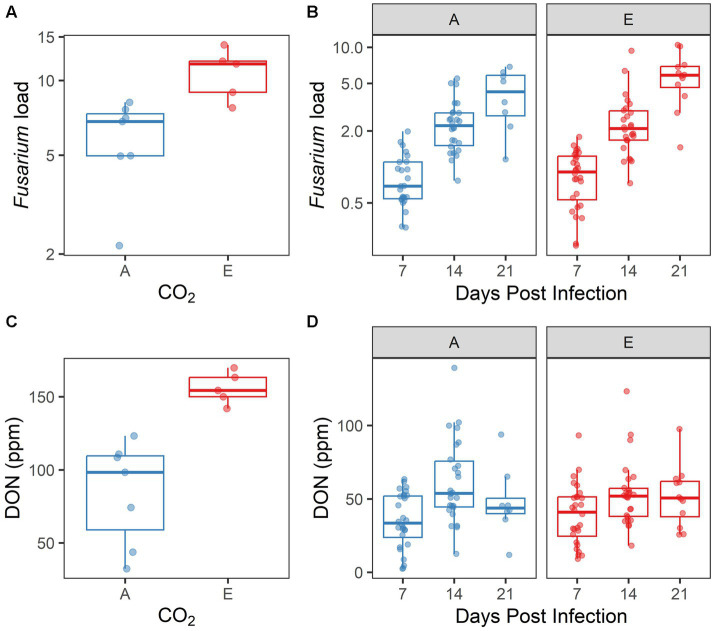
Disease symptoms induced by inoculated *Fusarium graminearum* vary by [CO_2_] treatment in **(A,C)** 2017, but not in **(B,D)** 2018. **(A,B)** *Fusarium* load and **(C,D)** DON accumulation, by CO_2_ treatment (A = ambient, E = elevated). In 2018 **(B,D)**, data are also shown by days post infection. Each point represents a single sample.

Within experimentally inoculated plants, we measured *Fusarium* load and [DON] as two metrics of disease progression. Both *Fusarium* load and [DON] differed significantly by [CO_2_] treatment in 2017 (*p* = 0.001, *p* = 0.004, respectively; Supplementary Table S5; Figures 5A,C). However, biological replication was low in this experiment because of spikes lost to herbivory, reducing the reliability of the 2017 data. A more robust dataset was produced in 2018, as herbivory was less problematic. In 2018, both *Fusarium* load and [DON] increased significantly over time (both *p* < 0.001; Supplementary Table S5; Figures 5B,D). However, neither *Fusarium* load nor [DON] were influenced by [CO_2_] treatment; nor was there an interaction between days post infection and [CO_2_] treatment for these response variables (all *p* > 0.10).

## 4. Discussion

Our data support the hypotheses that elevated [CO_2_] measurably impacts the structure of plant-associated microbiomes, and that relative abundances of individual microbial taxa, including economically important fungal pathogens, respond significantly to [CO_2_]. The impact of [CO_2_] on *Fusarium* and other microbiota was frequently contingent on tissue type and plant development (i.e., collection date). Manipulating [CO_2_] caused moderate shifts in the tissue-dependent communities in both years, with stronger temporal effects on community turnover in 2018. Our analyses of *Fusarium* spp. naturally found in wheat spikes contrasted with the result of our intentional *Fusarium* inoculation. Specifically, two strains of naturally occurring *Fusarium*, corresponding to two separate species complexes within the genus, showed opposing responses to [CO_2_]; while the inoculated *F. graminearum* strain GZ3639 showed no [CO_2_] response. Our results show that the response of crop-associated microbiomes to elevated [CO_2_] is complex and variable both within and across seasons.

Overall, the magnitude of microbiome changes induced by altered [CO_2_] in wheat was smaller than the differences observed in different plant tissue types (i.e., flag leaf vs. spike) or collection date. Similar findings have been reported in previous FACE studies. For example, Usyskin-Tonne et al. (2020) found that plant compartment (soil vs. root) had the greatest impact on belowground wheat bacterial communities, followed by plant age, then [CO_2_]. In our study, it is possible that microbial community responses to [CO_2_] were indirectly mediated by a plant phenological shift under elevated [CO_2_]. For example, elevated [CO_2_] has been shown to accelerate grain filling and senescence in wheat under certain environmental conditions (Li et al., 2001; Tun et al., 2021), but not others (Hay et al., 2022). However, we did not track phenological development closely, and it is also possible that there were other unmeasured co-occurring environmental changes created by the [CO_2_] manipulation. Similarly, the shift in microbiome communities under elevated [CO_2_] identified in both tissue types likely reflected the unique changes in those microbial habitats. Recent work shows that nutritional properties of wheat grain are often altered by elevated [CO_2_] (Hay et al., 2020), while foliar N is known to decrease dramatically under elevated [CO_2_] in C_3_ crops (Gonçalves et al., 2021).

Overall changes in microbiome community structure were also reflected by changes in individual microbial taxa. Proteobacterial taxa tended to be enriched over time under elevated [CO_2_], which matches previous reports in rice (Ikeda et al., 2015). However, differences between fungal phyla were more nuanced. Taxa belonging to the phylum Basidiomycota tended to experience more dramatic shifts in abundance between [CO_2_] treatments, relative to taxa from Ascomycota, but while some taxa were enriched under elevated [CO_2_], others were depleted. The responses of microbial taxa to elevated [CO_2_] may be dependent on the plant response to [CO_2_], such as immune response (Zhou et al., 2019), change in nutritional status (Hay et al., 2020; Gonçalves et al., 2021), or change in stomatal openings (Huang et al., 2018). Shifts in the abundance of individual microbial taxa may also depend on the positive and negative associations among the microbiota themselves (Connor et al., 2017).

Our results identified two strains of *Fusarium*, representing two distinct species complexes, that responded to [CO_2_] in 2018. The genus *Fusarium* has over 350 recognized species (Geiser et al., 2021), split across 23 species complexes. Several species complexes contain etiological agents capable of causing FHB in wheat (especially FSAMSC, FIESC, FTSC, and FFSC; Karlsson et al., 2021), with certain species complexes being more or less relevant to disease management, depending on region, climate and grain crop of interest (Xu and Nicholson, 2009). Most research in this area has focused on just one species, *F. graminearum*, although there have been a few studies of response to elevated [CO_2_] in other *Fusarium* species (e.g., *F. langsethiae*, *F. poae*, *F. pseudograminearum*, and *F. sporotrichioides*; Melloy et al., 2010; Kahla et al., 2023). Previous studies have shown that the impact of elevated [CO_2_] was dependent on both the strain and wheat cultivar identity (Cuperlovic-Culf et al., 2019), and the effect was due in part to variety-dependent changes in grain nutritional content (Hay et al., 2020). Specifically, the increased production of mycotoxins at elevated [CO_2_] was *F. graminearum* strain-dependent, but the results also correlated with cultivar-specific losses in grain protein and mineral content (Hay et al., 2020). When the FACE field wheat inoculation experiments described here were conducted, the authors were not yet aware of these potential strain- and cultivar-specific responses to [CO_2_]. Both the *F. graminearum* strain (Gz3639) and wheat cultivar (Glenn) used in this experiment have now been shown to be less responsive than some others are to elevated [CO_2_] (Cuperlovic-Culf et al., 2019; Hay et al., 2020, 2022). For these reasons, it is not surprising that no significant differences in FHB progression or in DON content were observed in our inoculation in 2018. Although effects of [CO_2_] on FHB progression and DON accumulation were suggested in 2017, less than 10% of the inoculated wheat heads were recovered due to herbivory; thus, those data were likely biased due to herbivore feeding preference.

A key strength of this study is that manipulation of [CO_2_] was accomplished under complex and realistic field conditions, compared to the more common experimental setting within controlled environment growth chambers. Due to the specialized infrastructure required for such an experiment, there have been few studies to date that have provided data on contemporary impacts of changing the composition of the atmosphere on crop-associated pathogens and microbiomes (Ainsworth and Long, 2021). Here, we studied the direct, contemporary effects of [CO_2_]. However, there are several downstream effects of elevated [CO_2_] that are also certain to impact plant-associated pathogens and microbiomes. For example, effects associated with climate change, such as patterns of precipitation and air temperatures, will also impact plant-microbe associations. Temperature has also been shown to influence the outcomes of interactions between wheat and *F. graminearum* (Hay et al., 2021). Of course, many of the impacts of climate change occur at larger geographic scales and across longer timeframes than can be studied within individual cropping seasons. Furthermore, pathogen populations can undergo rapid change due to selection and geographic mobility (Ward et al., 2008), and plant breeding continues apace; we will not be using today’s crop varieties under tomorrow’s climate scenarios. Nonetheless, attempting to simulate future environmental conditions remains the best available option for improving our ability to anticipate how forces of global change may create challenges for crop production and food safety.

Despite successfully manipulating [CO_2_] in a field setting, some artificiality remains in our study. In particular, the FACE infrastructure necessitated that some typically mechanized field operations (e.g., tilling and pesticide treatments) were performed manually, which may have had impacts on microbial transmissibility and colonization (Gdanetz and Trail, 2017). Additionally, due to constraints associated with other objectives of the FACE site, our wheat was embedded within a soybean field. Thus, the wheat plants likely experienced atypical plant–plant microbial transfer, versus what would be expected within a field planted entirely to wheat (Whitaker et al., 2022). Lastly, given the key experimental goal of evaluating [CO_2_]-induced FHB progression after inoculation, we only performed a more detailed assessment of naturally-infecting *Fusarium* differential abundance. While other putative pathogens likely exist in the dataset (e.g., *Bipolaris*), a full evaluation of these taxa was beyond the scope of this study.

The complexity of evaluating the impact of elevated [CO_2_] on plant-microbiome interactions in a field setting are highlighted by the differences between years. Although some confounded factors in the amplicon library preparation prevent our direct testing of differences between years, the responsiveness of microbiome structure to [CO_2_] likely varied between years due to differences in environmental conditions other than [CO_2_], such as temperature and precipitation (Ren et al., 2015). Infection and pathogenicity of *Fusarium* spp., in particular, are driven by moisture conditions during anthesis (Vaughan et al., 2016; Moraes et al., 2022). Accumulated precipitation in the summer months of 2018 was three-times that of 2017 (Prism Climate Group at Oregon State University, 2023), which may have contributed to our ability to detect differential abundance responses to [CO_2_] for the two naturally infecting strains (Verheecke-Vaessen et al., 2019).

In conclusion, our results describe how the wheat-associated microbiome and an economically important complex of cereal pathogens respond to experimental manipulation of [CO_2_] in a field setting. A key finding of this work is the demonstration of a link between [CO_2_] effects on the microbiome and collection date, which was likely the result of physiological changes in plant nutrient status and immune response over time (Cuperlovic-Culf et al., 2019; Hay et al., 2020; Rho et al., 2020; Gonçalves et al., 2021; Hay et al., 2022). A critical next step will be to assess how altered microbiomes under elevated [CO_2_] may lead to unforeseen consequences to crop yields or stress tolerance. Current microbiome studies are typically restricted to 16S and ITS markers for bacteria and fungi, respectively, which are less useful for strain level identification. However, the decades of genetic research on FHB-causing *Fusarium* spp. (Geiser et al., 2021) allowed us to show that *Fusarium* spp. response to elevated [CO_2_] was strain and species-complex specific. As sequencing technologies continue to advance, future studies may be better able to detect these strain-specific responses in other important genera of pathogens or plant growth promoting symbionts.

## Data availability statement

The datasets presented in this study can be found in online repositories. The names of the repository/repositories and accession number(s) can be found at: https://www.ncbi.nlm.nih.gov/, PRJNA544326.

## Author contributions

MB: Investigation, Writing – original draft, Conceptualization. BW: Formal analysis, Writing – original draft. SM: Investigation, Writing – review & editing. EA: Resources, Writing – review & editing. MV: Conceptualization, Investigation, Writing – review & editing.

## Footnotes

^1^ https://warm.isws.illinois.edu/warm/
