# Supplementary material for: Manipulating atmospheric CO2 concentration induces shifts in wheat leaf and spike microbiomes and in Fusarium pathogen communities
Source: Front Microbiol. 2023 Oct 10;14:1271219. doi: 10.3389/fmicb.2023.1271219 (PMC10595150; doi:10.3389/fmicb.2023.1271219)
Supplement: Supplementary file 2 [file Image_1.pdf]

Bakker MG, Whitaker BK, McCormick SP, Ainsworth EA and Vaughan MM (2023) Manipulating atmospheric CO_2 concentration induces shifts in wheat leaf and spike microbiomes and in *Fusarium* pathogen communities. Front. Microbiol. 14:1271219. doi: 10.3389/fmicb.2023.1271219

Supplementary Material

1 Supplementary Figures

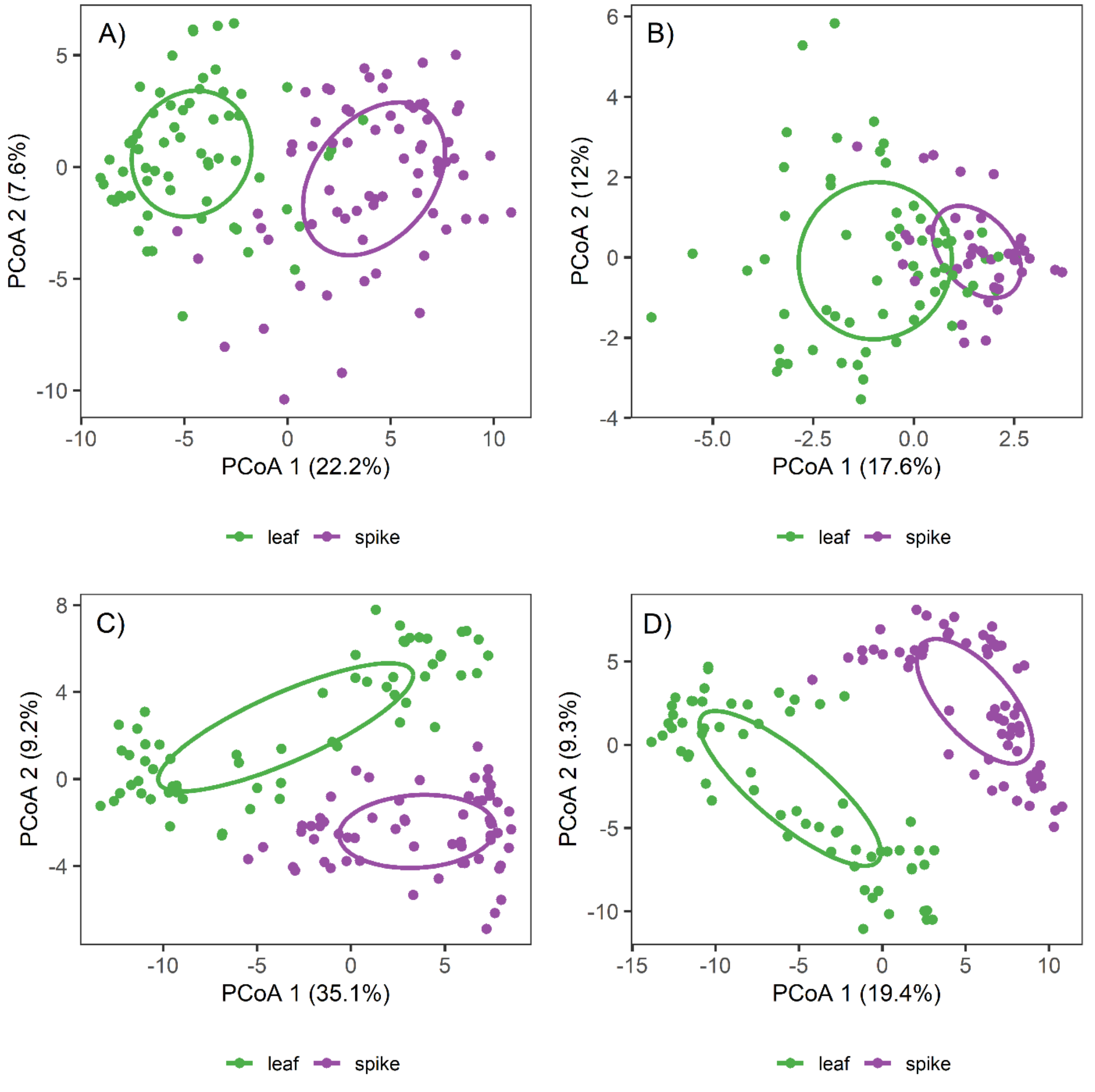

SI Fig. S1 – Microbiome structural differences by tissue type for: A) 2017 bacteria, B) 2017 fungi, C) 2018 bacteria, and D) 2018 fungi. Shown are principal coordinates ordinations based on pairwise Euclidean distances calculated from a variance stabilized matrix of amplicon sequence variant abundances. Each point represents a single sample. Ellipses represent one standard error around the centroid for each treatment group. Colors denote tissue types.

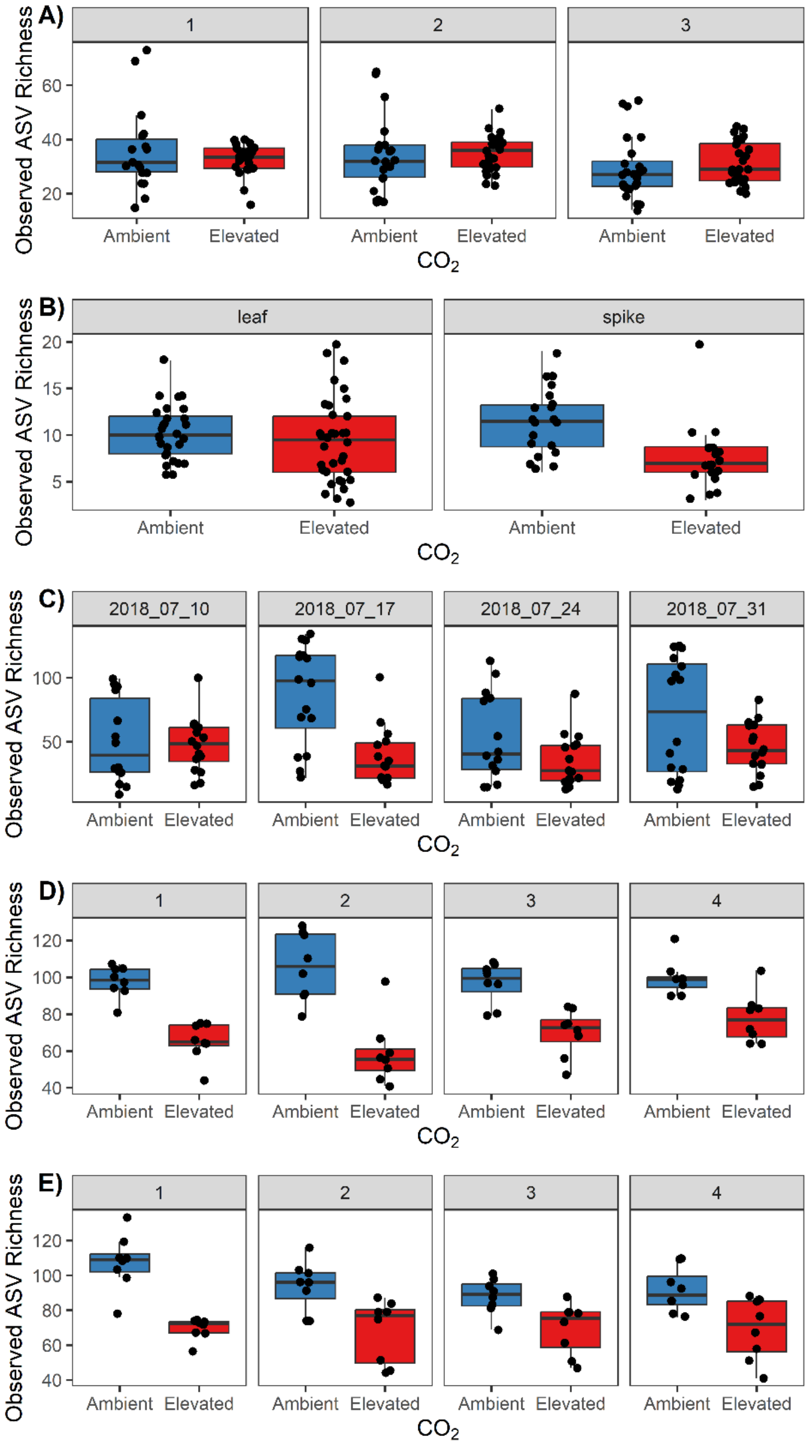

SI Fig. S2 – Observed microbial richness differences by treatment. Within each panel, differences in richness between ambient and elevated [CO_2] are shown on the x-axis. Each point represents a single sample. Panels depict: **A)** differences between block replicates for 2017 bacteria; **B)** tissue types for 2017 fungi; **C)** collection dates for 2018 bacteria; **D)** differences between block replicates for 2018 leaf fungi, **E)** differences between block replicates for 2018 spike fungi.

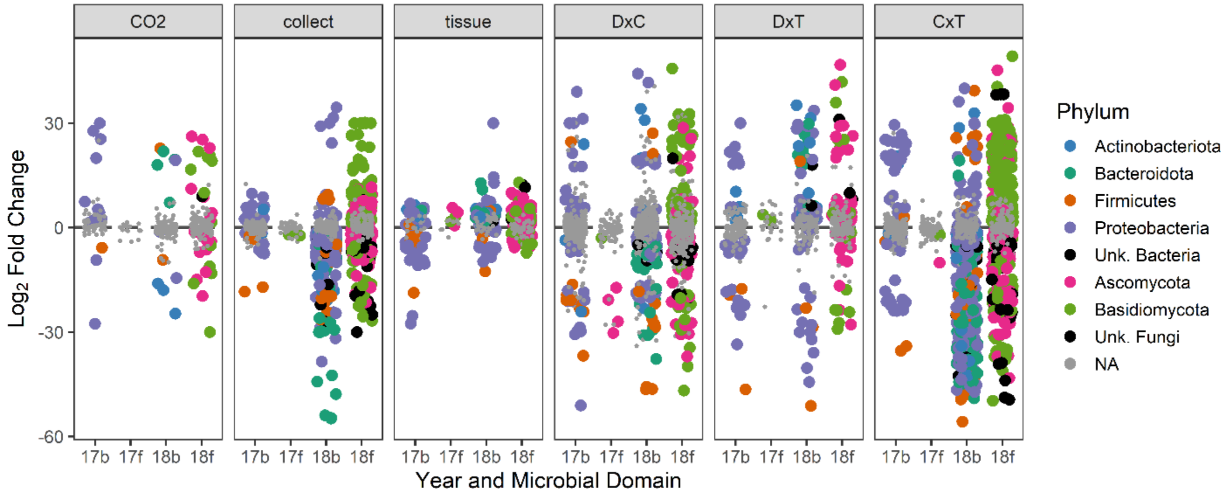

SI Fig. S3 – Taxonomic differences in $\log_2$ fold changes attributed to each experimental factor. Amplicon sequence variants that responded significantly to experimental factors are color coded by microbial phylum, while non-significant effects are shown in gray (see methods for full statistical details). ‘CO2’ = ambient vs. elevated concentration of carbon dioxide; ‘collect’ = collection date (3 dates in 2017, 4 dates in 2018), ‘tissue’ = spike vs. flag leaf, ‘D×C’ = interactions between carbon dioxide treatment and collection date, ‘D×T’ = interactions between carbon dioxide treatment and tissue type, ‘C×T’ = interactions between collection date and tissue type. 17b = bacteria in 2017; 17f = fungi in 2017; 18b = bacteria in 2018; 18f = fungi in 2018.

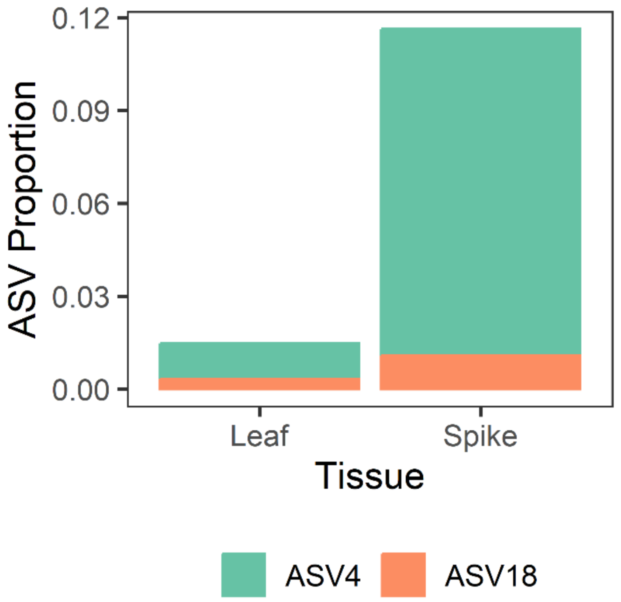

SI Fig. S4 – Two prevalent amplicon sequence variants (ASVs) assigned to *Fusarium* in 2018 are differentially abundant in spike versus flag leaf tissues. Differential abundance was indicated by DESeq2 (see methods for full statistical details). Bar plots depict the proportion of cumulative sequencing reads across all samples in each treatment group.
